# Sfp1 and Rtg3 reciprocally modulate carbon source‐conditional stress adaptation in the pathogenic yeast *Candida albicans*

**DOI:** 10.1111/mmi.13722

**Published:** 2017-06-19

**Authors:** Stavroula L. Kastora, Carmen Herrero‐de‐Dios, Gabriela M. Avelar, Carol A. Munro, Alistair J. P. Brown

**Affiliations:** ^1^ Aberdeen Fungal Group, MRC Centre for Medical Mycology, School of Medical Sciences, Institute of Medical Sciences University of Aberdeen Aberdeen AB25 2ZD UK

## Abstract

The pathogenicity of the clinically important yeast, *Candida albicans*, is dependent on robust responses to host‐imposed stresses. These stress responses have generally been dissected *in vitro* at 30°C on artificial growth media that do not mimic host niches. Yet host inputs, such as changes in carbon source or temperature, are known to affect *C. albicans* stress adaptation. Therefore, we performed screens to identify novel regulators that promote stress resistance during growth on a physiologically relevant carboxylic acid and at elevated temperatures. These screens revealed that, under these ‘non‐standard’ growth conditions, numerous uncharacterised regulators are required for stress resistance in addition to the classical Hog1, Cap1 and Cta4 stress pathways. In particular, two transcription factors (Sfp1 and Rtg3) promote stress resistance in a reciprocal, carbon source‐conditional manner. *SFP1* is induced in stressed glucose‐grown cells, whereas *RTG3* is upregulated in stressed lactate‐grown cells. Rtg3 and Sfp1 regulate the expression of key stress genes such as *CTA4, CAP1* and *HOG1* in a carbon source‐dependent manner. These mechanisms underlie the stress sensitivity of *C. albicans sfp1* cells during growth on glucose, and *rtg3* cells on lactate. The data suggest that *C. albicans* exploits environmentally contingent regulatory mechanisms to retain stress resistance during host colonisation.

## Introduction

Of the *circa* 1.5 million fungal species thought to inhabit our planet, only around 600 have been reported to be pathogenic for humans. The yeast *Candida albicans* is a common cause of mucosal infection (oral and vaginal thrush), and is the most frequent cause of nosocomial fungal infections (Brown *et al*., 2007; Brock, 2009; Calderone and Clancy, 2012). The fate of this opportunistic fungal pathogen is intertwined with its mammalian host, in which it is normally found as a relatively harmless commensal in the oral, urogenital and gastrointestinal microbiota (Bouza and Muñoz, 2008; Calderone and Clancy, 2012). However, infections can arise when our immunological defenses become compromised, allowing *C. albicans* to thrive in niches where it would normally be subject to phagocytic clearance (Gow *et al*., 2012; Brown *et al*., 2014a,).

The ability of *C. albicans* to colonise diverse host niches is dependent on its rapid adaptation to the local conditions in these microenvironments, including changes in the availability of key nutrients such as the carbon source (Staib *et al*., 1999; Barelle *et al*., 2006; Ene *et al*., 2013; Brown *et al*., 2014b). For example, glucose levels are minimal in the colon, between 0.06 and 0.1% in the bloodstream, and are reported to be about 0.5% in vaginal secretions (Brown *et al*., 2014b). Transcript profiling studies that have examined the *in vivo* gene expression patterns of *C. albicans* cell populations from the blood or internal organs suggest that both glycolytic and gluconeogenic pathways are active in these fungal populations (Andes *et al*., 2005; Fradin *et al*., 2005; Barelle *et al*., 2006; Walker *et al*., 2008). This counterintuitive finding could be explained either by an ability of individual *C. albicans* cells to express both pathways simultaneously (Sandai *et al*., 2012; Childers *et al*., 2016) or by the complexity of host niches, in which individual *C. albicans* cells can be exposed to glucose‐containing or glucose‐lacking microenvironments depending on their location (Hube, 2004; Barelle *et al*., 2006; Miramón *et al*., 2012). In the gut, most dietary sugars are absorbed in the small intestine before the remaining nutrients enter the large intestine. This view is supported by bacterial expression profiling studies, which suggest that sugar concentrations are minimal in colon microenvironments (Kröger *et al*., 2013; Avican *et al*., 2015). It has been reported that glycolytic genes are upregulated in *C. albicans* cells colonizing the mouse caecum (Rosenbach *et al*., 2010), but these experiments involved the pretreatment of mice with antibiotics to deplete the gut microbiota. Indeed, the view that sugars are limiting in gut microenvironments is reinforced by the observation that *C. glabrata* mutants that cannot utilize the organic acid lactate are unable to colonize the gut (Ueno *et al*., 2011).

The pathogenicity of *C. albicans* is further enhanced by its ability to counteract local environmental stresses. This yeast is relatively resistant to certain stresses compared with other fungi (Jamieson *et al*., 1996; Nikolaou *et al*., 2009). Host‐imposed stresses include oxidative, nitrosative and cationic stresses, as well as thermal fluctuations in febrile hosts (Enjalbert *et al*., 2003; Hube, 2004; Enjalbert *et al*., 2006; Enjalbert *et al*., 2007; Leach *et al*., 2012a,b,c; Miramón *et al*., 2012). *C. albicans* mounts robust responses to these stresses via specific signaling pathways. The Hog1‐dependent MAP kinase pathway promotes resistance to cationic, osmotic and oxidative stresses (San José *et al*., 1996; Alonso‐Monge *et al*., 2003; Smith *et al*., 2004). Additional MAP kinase signaling pathways, characterized by the Mkc1 and Cek1 MAP kinases, promote the resistance of *C. albicans* to cell wall stresses (Navarro‐García *et al*., 1995; Alonso‐Monge *et al*., 2006; Eisman *et al*., 2006). The AP‐1‐like transcription factor, Cap1, plays a major role in driving the transcriptional response to oxidative stress (Alarco and Raymond, 1999; Znaidi *et al*., 2009; Kos *et al*., 2016), and the response‐regulator Skn7 contributes to this response (Singh *et al*., 2004). Meanwhile, the transcription factors Cta4 and Hsf1 play key roles in the transcriptional responses to nitrosative stress and heat shock, respectively (Hromatka *et al*., 2005; Chiranand *et al*., 2008; Nicholls *et al*., 2009). These signaling pathways protect *C. albicans* against many of the stresses imposed by the host (Alonso‐Monge *et al*., 2006; Herrero‐de‐Dios *et al*., 2010). Consequently, the inactivation of key stress responses attenuates the virulence of this fungus (Wysong *et al*., 1998; Alonso‐Monge *et al*., 1999; Hwang *et al*., 2002; Fradin *et al*., 2005; Nicholls *et al*., 2011).

The *in vitro* dissection of these signaling pathways and their contributions to stress adaptation has generally been performed using *C. albicans* cells grown at 30°C on rich media containing 2% glucose. However, as described above, many host niches colonized by this pathogenic yeast contain low levels of glucose or lack this sugar. Also, *C. albicans* is subjected to changes in ambient temperature, in the febrile host for example. Therefore, during host colonization, *C. albicans* must respond to local environmental stresses while adapting to alternative carbon sources or thermal fluctuations. Changes in temperature or carbon source have been shown to affect the stress resistance of *C. albicans* cells. For example, ambient temperature influences their resistance to osmotic and cell wall stresses (Leach *et al*., 2012a). Exposure to glucose enhances the resistance of *C. albicans* to oxidative stress (Rodaki *et al*., 2009). Also, growth on lactate rather than glucose confers elevated resistance to osmotic stress (Ene *et al*., 2012a, 2012b; Ene *et al*., 2015).

These observations suggest crosstalk between carbon assimilation and stress adaptation in *C. albicans*, but the mechanisms that underlie this crosstalk remain to be defined. Therefore, we performed high‐throughput robotic screens to identify *C. albicans* mutants that display carbon source‐ or temperature‐conditional resistance to oxidative, osmotic or nitrosative stresses. Our screens, which have revealed extensive environmentally conditional stress sensitivities, have highlighted two transcription factors that play complementary roles in the carbon‐conditional modulation of stress responses. Rtg3 promotes stress adaptation in lactate‐grown cells, whereas Sfp1 enhances stress adaptation in glucose‐grown cells. Mechanisms such as these presumably allow *C. albicans* to maintain robust stress responses as it colonizes host microenvironments with different nutrient profiles.

## Results

### Carbon‐conditional stress sensitivity in *C. Albicans*

High‐throughput robotic screens of previously constructed mutant collections (Supporting Information Table S1) were used to identify genes that promote environmentally contingent stress adaptation in *C. albicans*. These collections included the set of regulatory transposon insertion mutants generated by the Mitchell laboratory (Norice *et al*., 2007; Nobile and Mitchell, 2009), the transcription factor deletion mutants constructed by Sanglard's group (Vandeputte *et al*., 2011), and the library of null mutants created by Noble and co‐workers (Noble *et al*., 2010). These mutants represent approximately 16% of *C. albicans* protein coding genes. The stress resistance of each mutant was compared to its congenic control strain using plate assays under a range of growth conditions. Moderate doses of oxidative (0.4 mM H_2_O_2_) and nitrosative stress (5 mM NaNO_2_) were used (Chiranand *et al*., 2008). We also used a relatively moderate dose of salt (1 M NaCl) (Kaloriti *et al*., 2012), which imposes osmotic and cationic stress (Hohmann, 2002). In control experiments these doses were shown to significantly attenuate the growth of hallmark deletion mutants, such as *hog1, cap1* or *cta4*, but not the growth of the corresponding wild type control strains under standard growth conditions (YPD at 30°C: Experimental Procedures). The stress sensitivities of all mutants were then compared in 96 array format on rich glucose‐containing medium and minimal media containing glucose or lactate as sole carbon source at 30°C, 37°C and 42°C (36 conditions in total, including the unstressed controls) (Supporting Information Fig. S1). The plates were then imaged, and the growth of each strain, relative to its isogenic wild type control, was recorded by computational analysis of these raw images. A strain was defined as displaying stress sensitivity if it consistently displayed more than an 80% reduction in growth in the presence of that stress relative to the control plate without the stress but with matching carbon and temperature conditions (Experimental Methods). The data were filtered to exclude mutants that were unable to grow under the control unstressed condition. The output was then used to construct carbon‐ and temperature‐conditional stress networks.

**Figure 1:**
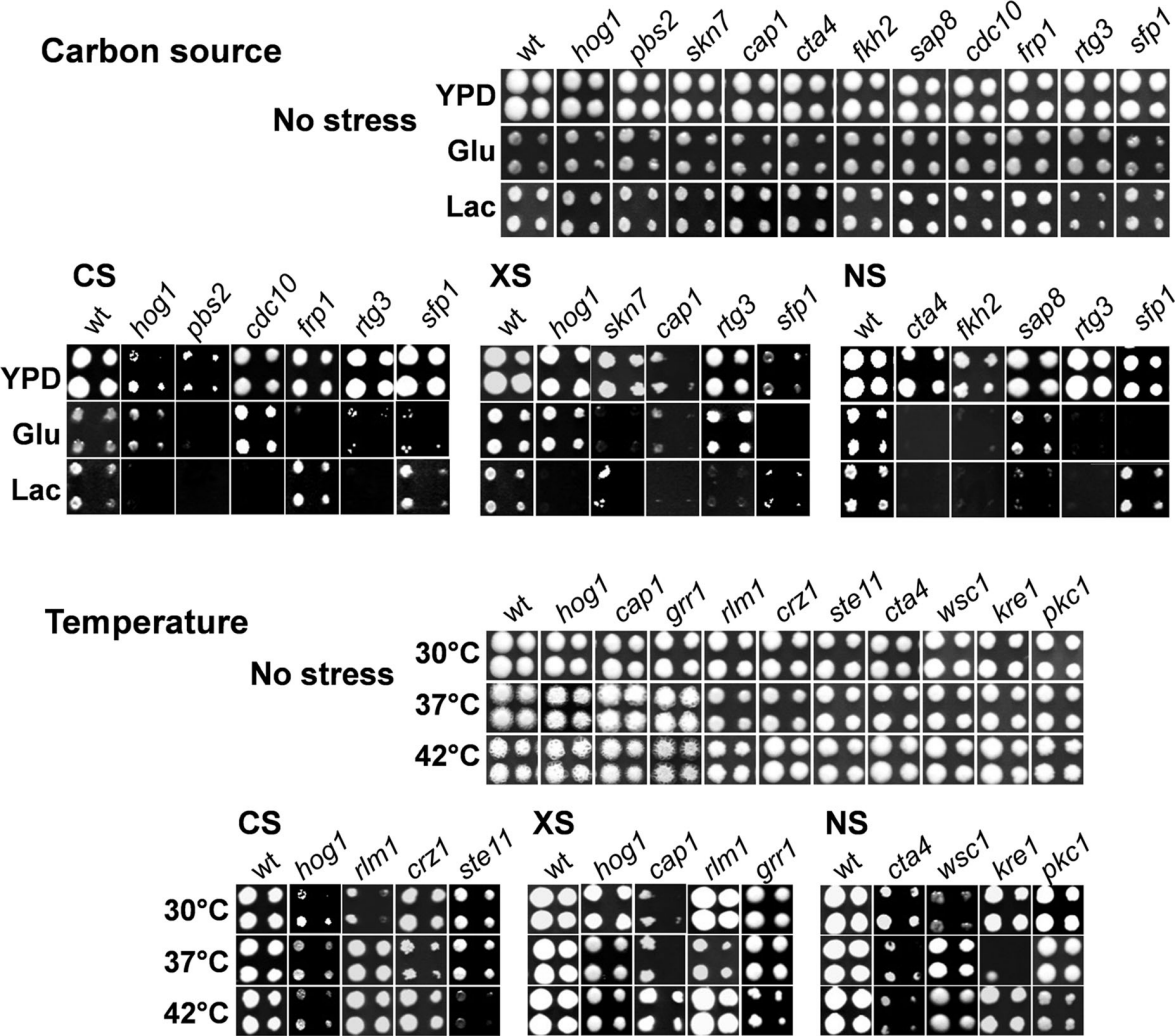
*C. albicans* mutants that display carbon‐ and temperature‐conditional stress sensitivity. To examine the impact of carbon source, *C. albicans* mutants were robotically plated onto YPD, GYNB (Glu) or LacYNB (Lac) containing cationic stress (CS; 1 M NaCl), oxidative stress (XS; 0.4 mM H_2_O_2_), nitrosative stress (NS; 5 mM NaNO_2_) or no stress, and grown at 30°C (Experimental Procedures). The robot generated four spots per strain: two at higher, and two at lower cell densities. To examine the impact of ambient temperature, *C. albicans* strains were plated on YPD and grown at the temperature indicated. Examplar strains are shown in this figure, whilst the full sets of environmentally contingent stress sensitive mutants are shown in Figs. 2 and 3.

**Figure 2:**
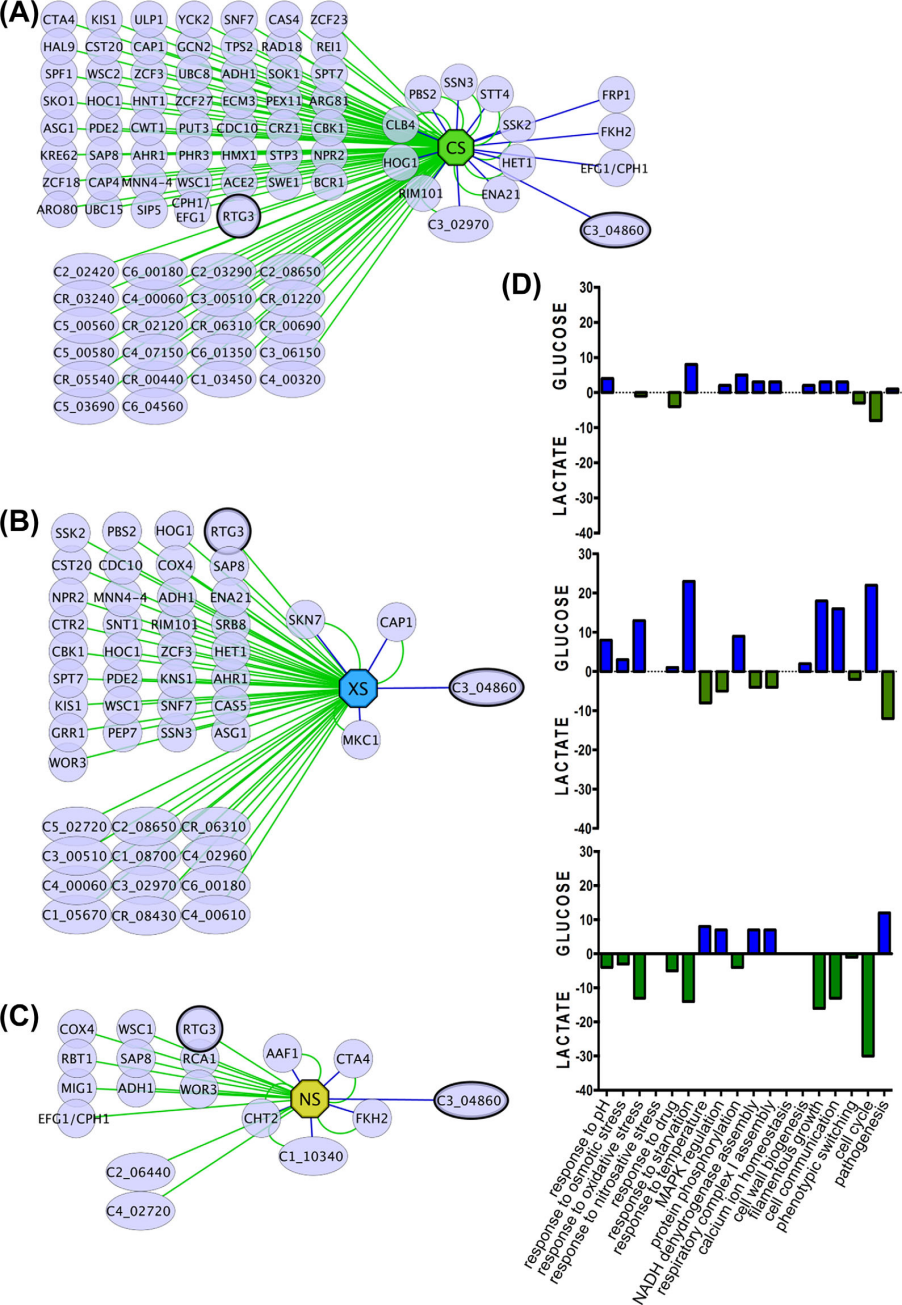
Networks of *C. albicans* mutants revealed by the screens for carbon‐conditional stress sensitivity. The networks of carbon‐conditional mutants (at 30°C) are displayed for: A. Cationic stress (CS, 1 M NaCl, green octagon). B. Oxidative stress (XS, 0.4 mM H_2_O_2_, blue octagon). C. nitrosative stress (NS, 5 mM NaNO_2_, yellow octagon) using edges between these hubs and the gene nodes that are either green (sensitivity on lactate) or blue (sensitivity on glucose). Nodes with double edges represent mutants with sensitivity on both carbon sources. Standard name genes are represented with circles and systematic name genes with diamonds. D. Significant enrichment of specific GO terms (biological processes) for lactate‐conditional (green) and glucose‐conditional mutants (blue).

Mutants with defects in the HOG signaling module did not display carbon conditional sensitivity to cationic stress: *pbs2* and *hog1* cells were sensitive to NaCl whether cells were growth on rich glucose‐containing medium (YPD) or on minimal medium containing glucose or lactate as sole carbon source (Fig. 1). Cells lacking Pbs2 seemed more sensitive to cationic stress than *hog1* cells when grown on glucose as sole carbon source (GYNB; Fig. 1), but in the context of our screen, both mutants displayed greater stress sensitivity than wild type cells under this growth condition. Therefore, this key MAP kinase module contributes to cationic stress adaptation under conditions outwith the standardized conditions that have generally been used to dissect *C. albicans* stress adaptation *in vitro*. However, our screens revealed many other *C. albicans* mutants that did display carbon‐conditional sensitivity to cationic stress. In comparison with glucose‐grown cells, cells growing on lactate required many additional functions for adaptation to cationic stress. This did not simply reflect the additional biochemical functions required for growth on lactate, because in our screen, the stress sensitivity of a mutant grown on lactate was defined in comparison to its growth on lactate in the absence of stress. The carbon‐conditional cationic stress sensitive mutants included *cdc10* (lacking a septin required for virulence and tissue invasion)*, frp1* (ferric reductase) and *sfp1* (C_2_H_2_ transcription factor involved in regulation of biofilm formation: Chen and Lan, 2015) (Fig. 1). Interestingly, both *cap1* and *cta4* cells were sensitive to cationic stress during growth on lactate, but not on glucose (Fig. 2A). Under standard growth conditions (YPD), Cap1 and Cta4 play central roles in the transcriptional responses to oxidative and nitrosative stresses, respectively *(*Alarco and Raymond, 1999; Chiranand *et al*., 2008; Znaidi *et al*., 2009). Our data suggest that these transcription factors also contribute to cationic stress adaptation under other growth conditions. Mutants with defects in cell wall biosynthesis (*crz1, kre62, mnn4, phr3*), cellular morphogenesis and biofilm formation (*efg1/cph1, ace2, bcr1, cbk1, cdc10, pde2, swe1, wsc1*) also displayed carbon conditional cationic stress sensitivity. These genes are required for normal levels of NaCl resistance during growth on lactate (Fig. 2A).

Other *C. albicans* mutants showed carbon‐conditional sensitivity to oxidative stress. These included *rtg3* (*C1_10990C*), which encodes a putative transcription factor orthologous to *Saccharomyces cerevisiae* Rtg3 and recently shown to be involved in galactose metabolism in *C. albicans* (Dalal *et al*., 2016), and also *sfp1* (*C3_04860W*), which as mentioned above, encodes a partially characterized transcription factor involved in biofilm formation (Fig. 1). Interestingly, *hog1* cells were sensitive to oxidative stress during growth on lactate, but not on minimal medium containing glucose (Fig. 1). This was also the case for *ssk2* and *pbs2* cells, suggesting that the HOG MAPK module makes environmentally contingent contributions to oxidative stress resistance (Fig. 2B). In contrast, *cap1* and *skn7* cells, which lack key players in the oxidative stress response under standard growth conditions, were sensitive to oxidative stress during growth on lactate as well as glucose. Therefore, Cap1 and Skn7 would appear to promote oxidative stress adaptation under conditions additional to the standardized *in vitro* growth conditions that have generally been used to examine these regulators (Alarco and Raymond, 1999; Zhang *et al*., 2000; Alonso‐Monge *et al*., 2003; Singh *et al*., 2004; Wang *et al*., 2006; Enjalbert *et al*., 2007).

Carbon‐conditional sensitivity to nitrosative stress was also displayed by some specific *C. albicans* mutants (Fig. 1). These included morphogenetic mutants such as *cph1/efg1* and *rbt1* (Fig. 2C). They also included the *rtg3* and *sfp1* mutants, which also displayed carbon‐conditional sensitivities to cationic and oxidative stresses (Fig. 1). Once again the significance of carbon‐conditional nitrosative stress phenotypes was emphasized by the observation that *cta4* cells, which lack the key regulator that drives transcriptional responses to nitrosative stress, displayed sensitivity to this stress during growth on glucose and lactate (Fig. 2C). Interestingly, mutants lacking Fkh2 (forkhead transcription factor involved in morphogenetic regulation), Cht2 (chitinase) or Aaf1 (adhesin‐like protein) were sensitive to nitrosative stress, irrespective of the carbon source. These findings reinforce the previously reported links between metabolic adaptation, cell wall biogenesis and morphogenesis (Ene *et al*., 2012a, 2012b; Brown *et al*., 2014a).

The carbon‐conditional stress mutants identified in our screens displayed significant enrichment of specific functional categories (GO terms), relative to the functional categories represented in the entire mutant set used in the screens (Fig. 2D). For example, genes related to ‘Pathogenesis’ were enriched in lactate‐dependent oxidative stress genes, and genes related to ‘Filamentous Growth’ were enriched in lactate‐dependent nitrosative stress genes (Fig. 2D). A significant proportion of the carbon‐conditional stress mutants we identified carry defects in uncharacterized genes (33% of the carbon‐conditional cationic stress mutants) (Fig. 2A). This reflects our ignorance about the ways in which environmental changes within host niches impact upon stress adaptation mechanisms in this major pathogen.

### Temperature‐conditional stress sensitivity in *C. Albicans*

**Figure 3:**
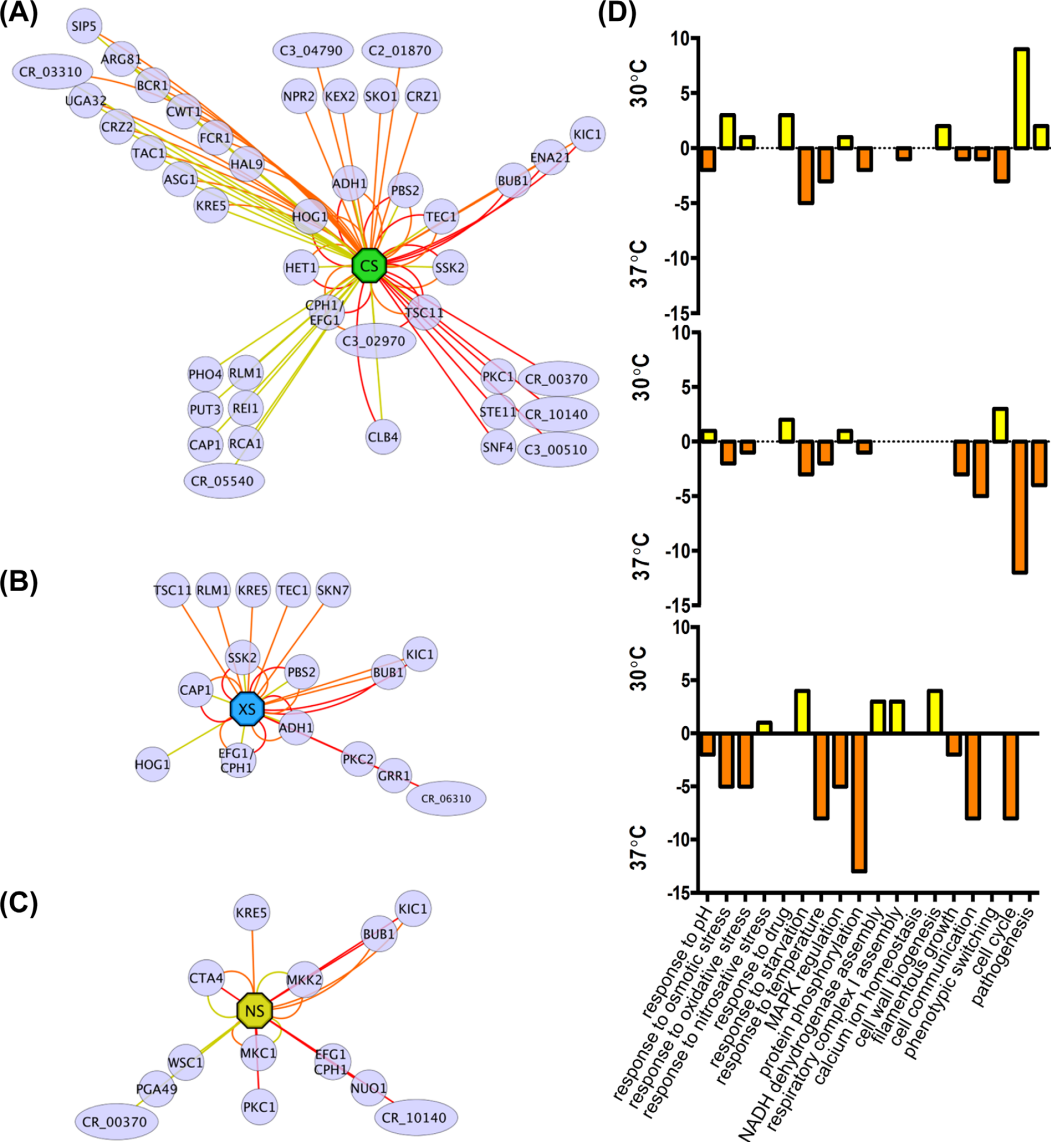
*C. albicans* gene networks revealed by screens for temperature‐conditional stress sensitivity. The networks of temperature‐conditional mutants (on YPD) are displayed for: A. cationic stress (CS, 1 M NaCl, green octagon) B. oxidative stress (XS, 0.4 mM H_2_O_2_, blue octagon) C. nitrosative stress (NS, 5 mM NaNO_2_, yellow octagon) using edges between these hubs and gene nodes that are either yellow (sensitivity at 30°C), orange (sensitivity at 37°C) or red (sensitivity at 42°C). Nodes with double or triple edges indicate stress sensitivity at two or more temperatures. Standard name genes are represented with circles and systematic name genes with diamonds. D. Significant enrichment of specific GO terms (biological processes) at 30°C (yellow) and 37°C (orange).

Our screens also revealed numerous *C. albicans* mutants that display temperature‐conditional stress phenotypes. For example, the sensitivity of *rlm1* cells to cationic and oxidative stress was higher at 30°C than at 37°C and 42°C. In contrast, *crz1* cells were more sensitive to cationic stress at 37°C (Fig. 3). The Rlm1 and Crz1 transcription factors promote cell wall remodelling in *C. albicans*, and the cell wall provides some protection against environmental insults. Therefore, the temperature‐conditional stress phenotypes of *rlm1* and *crz1* cells might suggest that Rlm1 and Crz1 make differential thermally contingent contributions to cell wall remodelling in *C. albicans* (Leach *et al*., 2012c), for example during caspofungin exposure (Walker *et al*., 2008). Both Rlm1 and Crz1 promote resistance to this antifungal drug *in vitro* (Hahn and Thiele, 2002; Selvaggini *et al*., 2004; Lesage and Bussey, 2006).

Mutants with defects in the HOG pathway (*ssk2, pbs2, hog1*) did not display temperature conditional cationic stress sensitivity (Fig. 3A). Furthermore, *cap1* cells showed no temperature conditionality in their oxidative stress sensitivity (Fig. 3B), and the *cta4* mutant did not display temperature conditional nitrosative stress sensitivity (Fig. 3C). These observations are consistent with the view that the Hog1, Cap1 and Cta4 signalling pathways play critical roles in cationic, oxidative and nitrosative stress adaptation, respectively, at ambient temperatures associated with host colonisation and invasion. However, *hog1* cells did not display significant oxidative stress sensitivity at 37°C or 42°C (Fig. 3C), which was consistent with the observation that Hog1 activation is reduced at higher temperatures in *C. albicans* (Smith *et al*., 2004), and that Hog1 is an Hsp90 client protein (Hawle *et al*., 2007; Diezmann *et al*., 2012). This might suggest that Hog1 plays a minor role in oxidative stress adaptation *in vivo*. In contrast, *skn7* cells were more sensitive to oxidative stress at 37°C, suggesting that Skn7 could play a greater role during host colonisation and invasion than might be predicted based on *in vitro* analyses performed at 30°C (Singh *et al*., 2004).

Temperature‐conditional stress mutants displayed significant enrichment in specific functional categories (Fig. 3D). As might be expected, ‘Temperature Stimulus’ genes were enriched in those mutants that displayed elevated stress sensitivity at 37°C, irrespective of the type of stress examined. In contrast, ‘Pathogenesis’‐related genes were enriched in the subsets of mutants that were sensitive to oxidative and nitrosative stress at 37°C, whereas ‘Pathogenesis’ genes were enriched in mutants that were cationic stress sensitive at 30°C. This observation highlights the significance of oxidative and nitrosative stresses to *C. albicans* cells *in vivo*.

### Environmental contingency of classical stress modules

**Figure 4:**
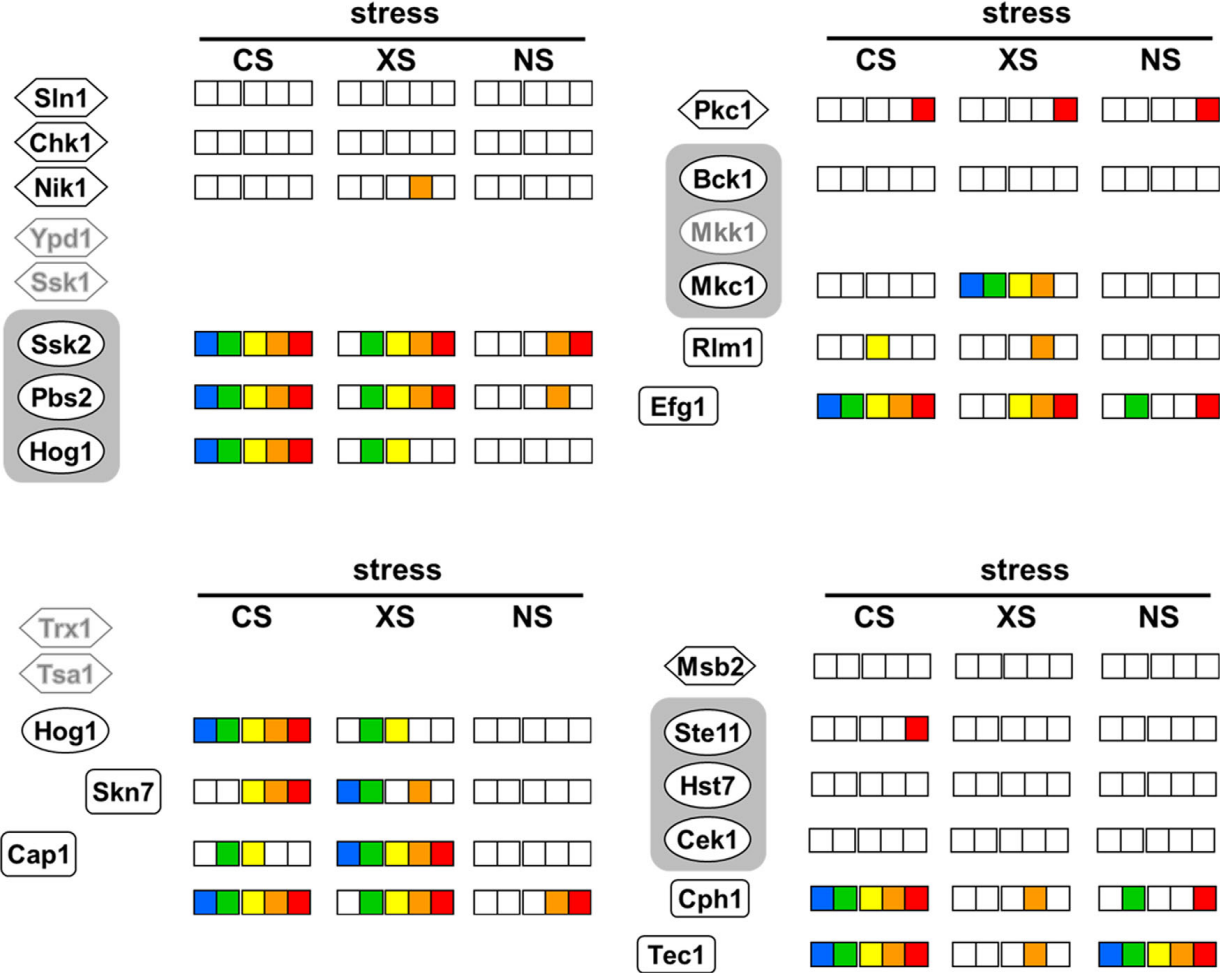
Temperature‐ and carbon‐conditional stress sensitivities for components of major stress pathways in *C. albicans*. Components of the Hog1 (top left quarter of the figure), Mkc1 (top right), Cap1 (bottom left) and Cek1 pathways (bottom right) are examined. On each pathway, those components for which cationic (CS), oxidative (XS) and nitrosative stress (NS) screening data are available are each named in black on the left, and have panels of boxes on the right: from left to right, sensitivity on glucose (blue), on lactate (green), at 30°C (yellow), 37°C (orange) or at 42°C (red). If no stress sensitivity was observed under a particular growth condition, then the corresponding box in the panel is white. MAP kinase modules are highlighted by rounded grey rectangles. Those components for which stress screening data were not available are named in grey and have no panels of boxes.

The carbon‐ and temperature‐conditional stress networks (Figs. 2 and 3) suggested that key stress signalling pathways might display differential contributions to stress resistance under certain growth conditions. To better illustrate this we mapped condition‐dependent stress sensitivities against four key pathways: the Cap1, Hog1, cell integrity (Mkc1) and hyphal MAP kinase (Cek1) pathways (Fig. 4). This exercise clearly showed that the Hog1 MAP kinase module (Ssk2, Pbs2, Hog1) is essential for cationic stress adaptation under all of the conditions analyzed. However, the Hog1 module contributes more to oxidative stress resistance during growth on lactate and at 30°C, than during growth on glucose or at 37°C or 42°C. Meanwhile, Cap1 contributes to oxidative stress resistance under all of the growth conditions tested, but contributes to cationic stress resistance in a carbon‐ and temperature‐conditional manner. Few components of the cell integrity pathway displayed significant stress sensitivity under the conditions tested, although *mkc1* cells displayed oxidative stress sensitivity under most of these conditions. Surprisingly, cells lacking the morphogenetic regulator Efg1 (Stoldt *et al*., 1997) displayed cationic stress sensitivity and oxidative stress sensitivity on rich, but not minimal growth media. With respect to the hyphal MAP kinase (Cek1) pathway, Cph1 and Tec1 displayed cationic stress sensitivity under all conditions tested, and interestingly, temperature‐conditional oxidative stress sensitivity. Under some conditions, certain components on each pathway were required for stress resistance whilst others were not (Fig. 4). This might reflect differences in the relative contribution of a signalling module or transcription factor to stress resistance under a particular growth condition. Nevertheless, taken together as a whole, the data indicate that these well‐studied signalling pathways mediate environmentally contingent outputs that are likely to have relevance *in vivo* during infection, but that have not been subjected to detailed examination *in vitro* so far.

### Complementary *Rtg3* and *Sfp1* regulons in *C. Albicans*

**Figure 5:**
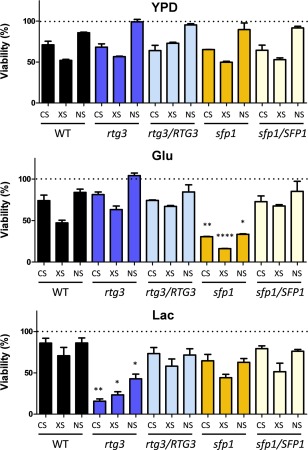
Carbon‐conditional contributions of *RTG3* and *SFP1* to stress resistance. The susceptibility of *rtg3* and *sfp1* mutants to cationic (CS), oxidative (XS) and nitrosative stress (NS) during growth on YPD, or on glucose (GLC) or lactate (LAC) as sole carbon source at 30°C. Percentage viability relative to the unstressed controls is presented. Statistically significant differences between the *rtg3* (blue) and *sfp1* (yellow) mutants and their corresponding reintegrant control strains (pale blue and pale yellow, respectively) are highlighted: *, *P* < 0.05; **, *P* < 0.01; ***, *P* < 0.001***; ****, *P* < 0.0001. Both the mutant and reintegrant strains were transformed with CIp30 to repair their remaining auxotrophies and to prevent *URA3* position effects (see Experimental Procedures).

Two mutants, namely *rtg3* and *sfp1*, appeared to display complimentary carbon‐conditional stress sensitivities. The *rtg3* mutant appeared more sensitive to cationic, oxidative and nitrosative stresses when grown on lactate, whereas *sfp1* cells were more sensitive to these stresses during growth on glucose (Fig. 1). Rtg3 is a bZIP transcription factor that is thought to be involved in galactose metabolism, cationic stress resistance, antifungal drug resistance and filamentous growth (Inglis *et al*., 2012; Yan *et al*., 2014; Dalal *et al*., 2016). Its orthologue in *S. cerevisiae* is a downstream effector of the TOR (Target of Rapamycin) pathway, which regulates growth in response to nutrients. Sfp1 is predicted to be a C_2_H_2_ transcription factor, the expression of which is induced in the rat catheter biofilm model (Nett *et al*., 2009; Inglis *et al*., 2012; Chen and Lan, 2015). In *S. cerevisiae*, Sfp1 regulates ribosomal protein gene transcription as well as responses to nutrients and stress (Inglis *et al*., 2012). On this basis we reasoned that *C. albicans* Rtg3 and Sfp1 might act to modulate stress responses in a complimentary carbon‐conditional manner (Fig. 1). Before pursuing this idea further we confirmed the carbon‐conditional stress sensitivities of *rtg3* and *sfp1* cells by comparing them against control reintegrant strains. The cationic, oxidative and nitrosative stress sensitivities of lactate‐grown *rtg3* cells were suppressed by reintroduction of the wild type *RTG3* gene. Also, the stress sensitivities of glucose‐grown *sfp1* cells were suppressed by transformation with wild type *SFP1* (Fig. 5). This confirmed that *rtg3* and *sfp1* cells display complimentary carbon‐conditional stress sensitivities.

**Figure 6:**
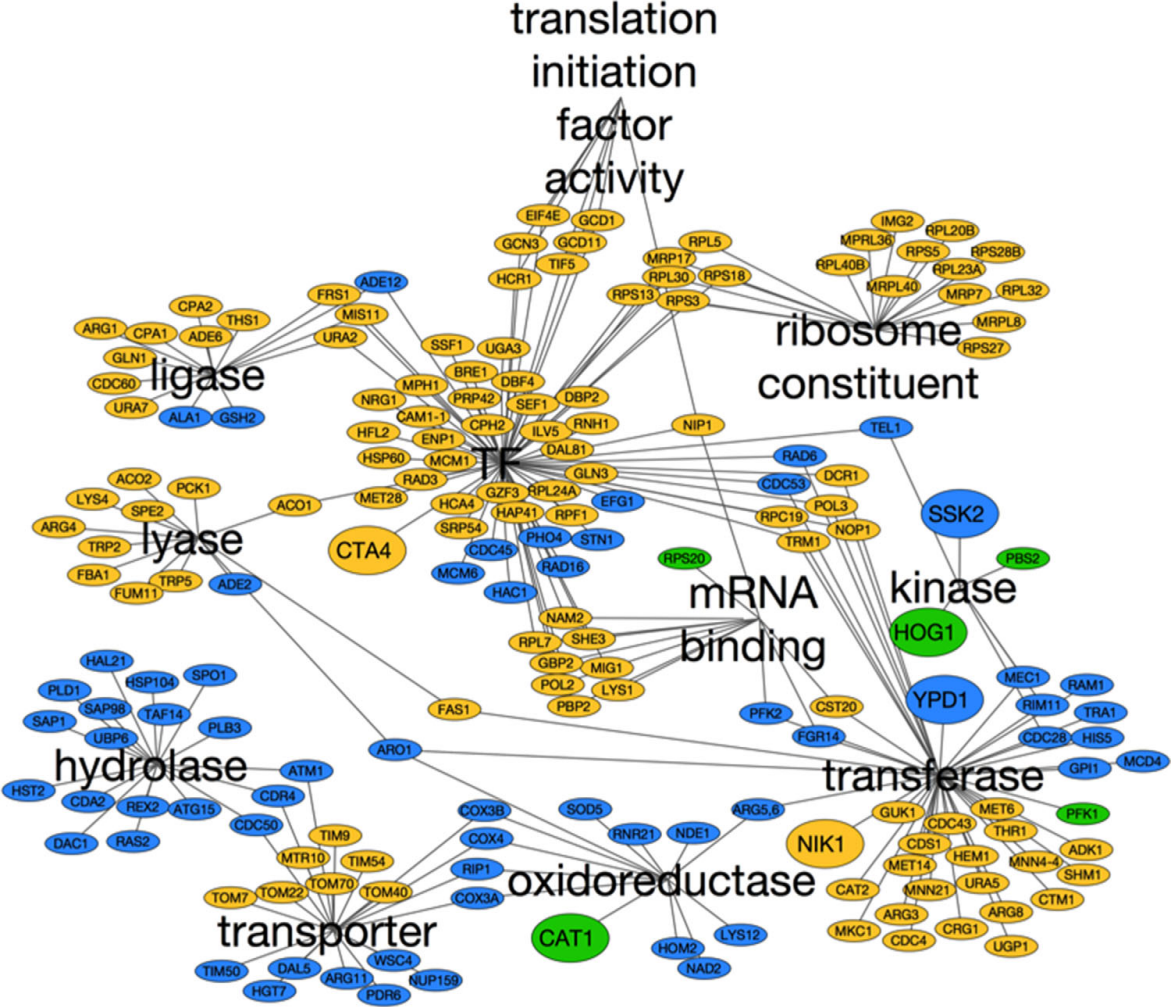
Rtg3 and Sfp1 regulons in *C. albicans*. Networks of putative gene targets of Rtg3 (blue) and Sfp1 (yellow) based on the presence of their consensus binding sites in the promoters of these genes. Those genes that might be targets for both transcription factors are highlighted in green. The networks are organized into significantly enriched GO categories (TF, transcription factors).

We then performed a bioinformatic analysis of putative Rtg3 and Sfp1 transcriptional target genes in *C. albicans* based on the presence of consensus Rtg3 (5′‐GTCACGT‐3′) or Sfp1 binding sites (5′‐AAA(A/T)TTT‐3′) in their promoter regions (Zhu *et al*., 2009; Perez *et al*., 2013). Genes encoding other transcription factors, translation and ribosomal proteins, kinases, oxidoreductases and transporters were amongst those identified using this approach (Fig. 6). Interestingly, this analysis suggested that Rtg3 and Sfp1 regulate complementary sets of genes in these functional categories. The small number of genes that may be regulated by both transcription factors include *HOG1, PBS2* and *CAT1* (catalase) (Fig. 6). Therefore, Rtg3 and Sfp1 are predicted to control complementary regulons involved in *C. albicans* growth and stress adaptation.

### *RTG3* and *SFP1* display carbon‐conditional stress induction

**Figure 7:**
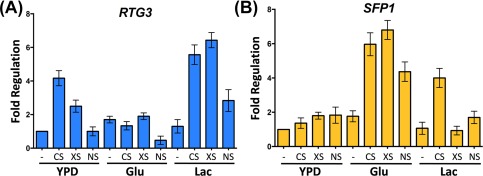
Carbon‐conditional induction of *RTG3* or *SFP1* genes in response to stresses. Induction of the *RTG3* (A) and SFP1 (B) transcripts (relative to the unstressed YPD control) after 10 min of exposure to cationic, oxidative or nitrosative stress.

We reasoned that differential *RTG3* and *SFP1* expression patterns might contribute to the complementary carbon‐conditional stress sensitivities of *rtg3* and *sfp1* cells. Therefore, using qRT‐PCR, we tested whether *RTG3* and *SFP1* transcript levels are induced in response to stress in wild type cells grown on lactate or glucose. Interestingly, the *RTG3* mRNA was induced in response to cationic, oxidative and nitrosative stress, but only in cells grown on lactate (Fig. 7A). In contrast, the *SFP1* mRNA was up‐regulated in response to cationic, oxidative and nitrosative stress in glucose‐grown cells. *SFP1* expression was induced in lactate‐grown cells following exposure to cationic stress, but not oxidative and nitrosative stress (Fig. 7B). Therefore, with only one exception (the cationic stress‐mediated induction of *SFP1* in lactate‐grown cells), *RTG3* and *SFP1* display complementary carbon‐conditional stress induction patterns that match the complementary carbon‐conditional stress sensitivities of *rtg3* and *sfp1* cells (Fig. 5).

### Rtg3 and Sfp1 control key stress genes in carbon‐conditional manner

**Figure 8:**
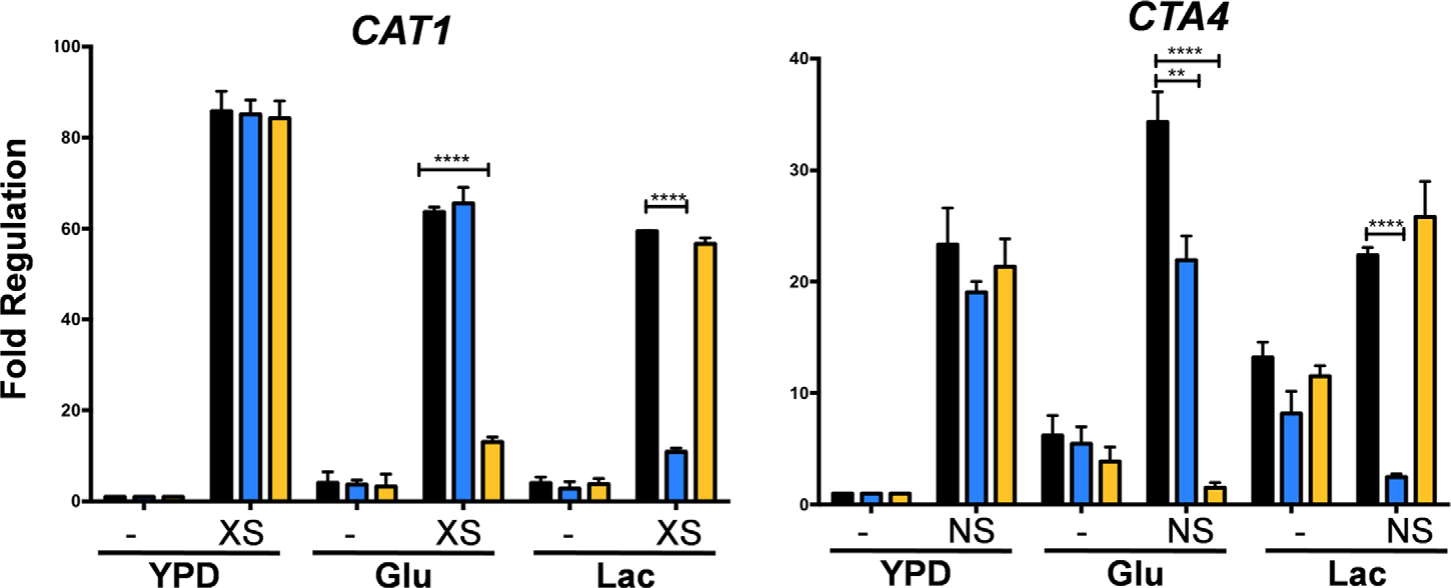
Impact of *RTG3* or *SFP1* inactivation upon the regulation of key oxidative and nitrosative stress genes in *C. albicans*. The levels of the *CAT1* transcripts were measured by qRT‐PCR, relative to the internal *ACT1* mRNA control, after 10 min of exposure to oxidative stress (XS) during growth on YPD, glucose or lactate: wild type, black; *rtg3*, blue; *sfp1*, yellow. Fold regulation was then calculated by normalizing *CAT1* transcript levels to those on YPD in the absence of stress. Using analogous procedures, *CTA4* transcript levels were measured in wild type, *rtg3* and *sfp1* cells following nitrosative stress (NS). Data represent the means and standard deviations from three independent experiments: *, *P* < 0.05; **, *P* < 0.01; ***, *P* < 0.001***; ****, *P* < 0.0001.

**Figure 9:**
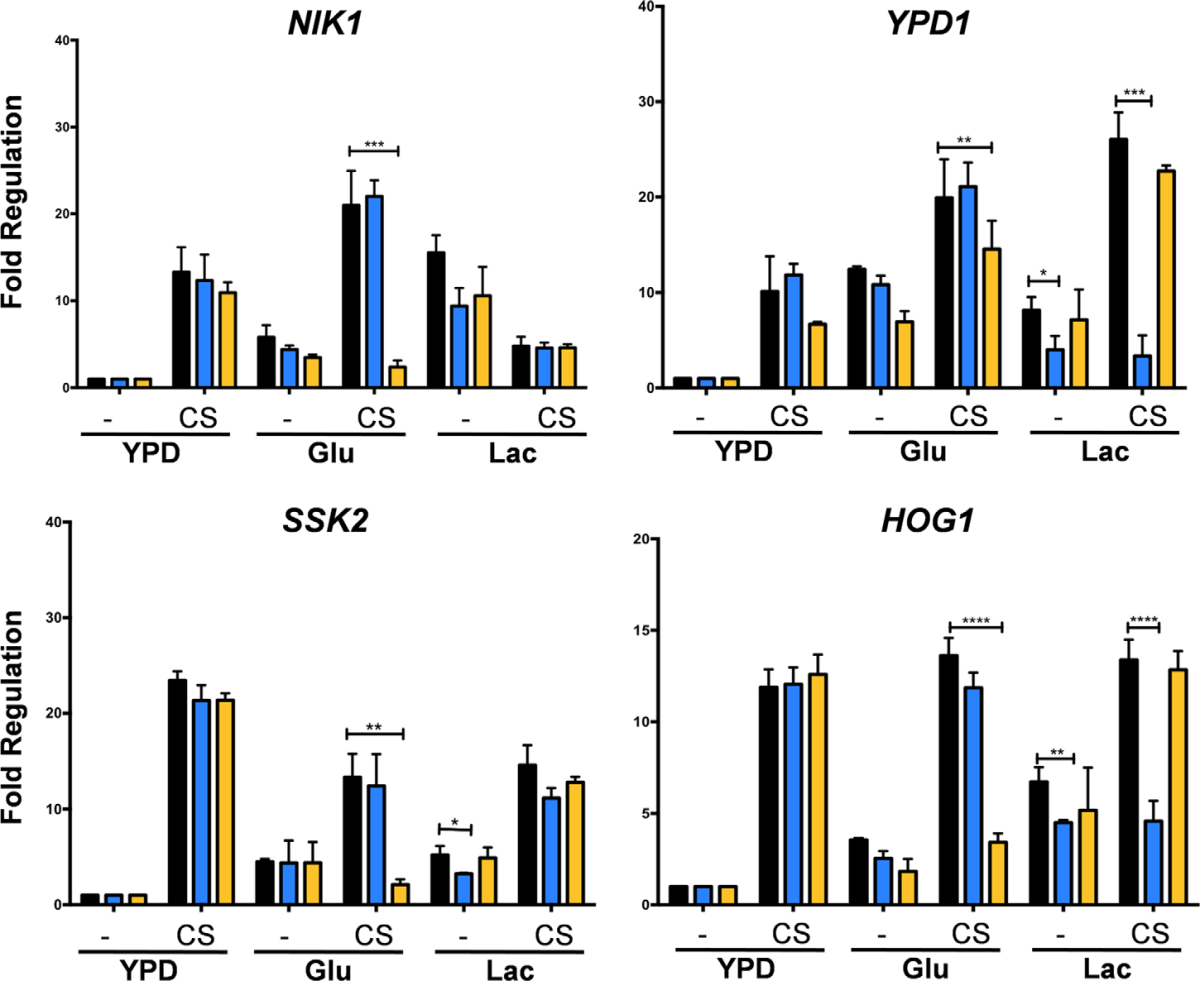
Impact of *RTG3* or *SFP1* inactivation upon the regulation of key cationic stress genes in *C. albicans*. The levels of the *NIK1, YPD1, SSK2* and *HOG1* transcripts were measured by qRT‐PCR, relative to the internal *ACT1* mRNA control, after 10 min of exposure to cationic stress (CS), during growth on YPD, glucose or lactate: wild type, black; *rtg3*, blue; *sfp1*, yellow. Fold regulation was then calculated by normalizing transcript levels to those on YPD in the absence of stress. Data represent the means and standard deviations from three independent experiments: *, *P* < 0.05; **, *P* < 0.01; ***, *P* < 0.001***; ****, *P* < 0.0001.

We then tested the impact of Rtg3 and Sfp1 on the expression of genes encoding key stress regulators in *C. albicans* that carry Rtg3 and Sfp1 consensus sequences in their promoter regions: *CAT1, CTA4, NIK1, YPD1, SSK2, PBS2* and *HOG1*. The levels of these transcripts were measured by qRT‐PCR in untreated and stressed wild type, *rtg3* and *sfp1* cells growing in YPD or in minimal medium containing glucose or lactate as sole carbon source. Both Rtg3 and Sfp1 were found to play major roles in the regulation of these genes in response to stress (Figs. 8 and 9).

In wild type cells, *CAT1* expression was strongly induced in response to oxidative stress under all three growth conditions (Fig. 8). The inactivation of Rtg3 or Sfp1 did not affect the up‐regulation of *CAT1* in cells grown on rich media (YPD). However, *CAT1* induction was significantly reduced in lactate‐grown *rtg3* cells and in glucose‐grown *sfp1* cells (Fig. 8). Therefore, the transcription factors Rtg3 and Sfp1 play complementary roles, up‐regulating *CAT1* expression in response to oxidative stress in cells growing on different carbon sources.

Similar observations were made for *CTA4* in the context of nitrosative stress (Fig. 8). In the absence of stress, basal *CTA4* transcript levels were higher in cells grown on minimal medium than in rich medium. Also, modest *CTA4* up‐regulation (about two‐fold) was observed following nitrosative stress treatment in lactate‐grown cells. Nevertheless, *CTA4* was upregulated by nitrosative stress under all growth conditions analysed. Interestingly, *CTA4* induction was significantly attenuated in lactate‐grown *rtg3* cells and in glucose‐grown *sfp1* cells (Fig. 8), reflecting the responses of *CAT1* to oxidative stress (Fig. 8). Therefore, Rtg3 and Sfp1 also play complementary roles in regulating *CTA4* expression in response to nitrosative stress.

We then analysed the regulation of *NIK1, YPD1, SSK2* and *HOG1* in response to cationic stress. The basal levels of these transcripts varied between cells grown on rich or minimal medium, but almost without exception, these mRNAs were induced in response to cationic stress in wild type cells grown in YPD, glucose or lactate (Fig. 9). The only exception was *NIK1*, which was not induced in NaCl‐treated lactate‐grown cells (Fig. 9). Significantly, the *NIK1, SSK1* and *HOG1* transcripts were not induced in NaCl‐treated *sfp1* cells growing on glucose. Also, the *YPD1* and *HOG1* mRNAs were not up‐regulated in NaCl‐treated *rtg3* cells grown on lactate (Fig. 9). Therefore, Rtg3 and Sfp1 play complementary carbon‐conditional roles in regulating the expression of key regulators of the cationic stress response in *C. albicans*.

## Discussion

The success of *C. albicans* as a pathogen is dependent on its ability to adapt to multifarious environmental challenges and cues in host niches. Our data support the view that the responses of *C. albicans* to some key environmental cues – stresses and nutrients – are tightly coordinated (Rodaki *et al*., 2009; Ene *et al*., 2012a,b). In addition to those regulators that have been shown to contribute to stress adaptation under standardized growth conditions *in vitro* (Fig. 4), we have demonstrated that many additional factors contribute to stress adaptation under alternative growth conditions (Figs. 1, 2, 3). This is particularly important because the standardized growth conditions that have generally been used to dissect stress responses in this pathogenic yeast (YPD at 30°C) do not accurately reflect host niches, where glucose is often limiting and the ambient temperature often approximates to 37°C. Our robotic screens of approximately 16% of *C. albicans* genes revealed novel regulators that promote *C. albicans* stress resistance in a carbon source‐ and temperature‐conditional manner (Figs. 1, 2, 3, 4). Many of these novel regulators are uncharacterised transcription factors (Fig. 2). This suggests that much remains to be discovered about the mechanisms that underlie environmentally contingent stress adaptation in this pathogen. This is significant because these mechanisms are likely to promote the physiological robustness of *C. albicans* in host niches, and hence could conceivably present novel targets for therapeutic intervention.

**Figure 10:**
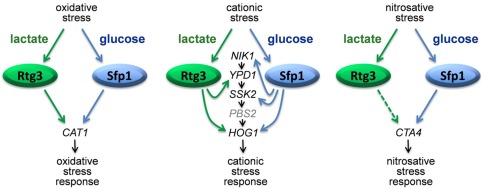
Model illustrating the carbon‐dependent modulation of stress adaptation in *C. albicans* by Rtg3 and Sfp1. Continuous arrows suggest direct effects of Rtg3 and Sfp1 upon gene expression, while dashed arrows suggest indirect effects (because a perfect match to the consensus binding site was not observed in the promoter of the target gene).

We then focussed on two regulators, Rtg3 (C1_10990c) and Sfp1 (C3_04860w), the inactivation of which caused complementary carbon‐conditional stress sensitivities in *C. albicans*. *Rtg3* cells were sensitive to cationic, oxidative and nitrosative stresses during growth on lactate, whereas *sfp1* cells were stress sensitive during growth on glucose (Figs. 1 and 5). The expression of *RTG3* and *SFP1* was induced in lactate‐ and glucose‐grown cells, respectively (Fig. 7), which was consistent with the carbon‐conditional stress sensitivities of *rtg3* and *sfp1* cells. Many *C. albicans* transcription factors bind similar DNA sequences to their *S. cerevisiae* orthologues (e.g. Tripathi *et al*. 2002; Nicholls *et al*., 2009, 2004; Ihmels *et al*., 2005; Tsong *et al*., 2006). This has been confirmed for Rtg3 (Perez *et al*., 2013), but not for Sfp1. Nevertheless, on this basis, Rtg3 or Sfp1 appear to regulate complementary sets of genes with related functions, such as transcription factors, kinases, transporters, transferases and ligases (Fig. 6). This does not simply reflect the nature of the mutants in the libraries that were screened because these represent many other functional categories (e.g. Figs. 2 and 3). We then showed that Rtg3 and Sfp1 regulate the expression of genes encoding key regulators of the cationic, oxidative and nitrosative stress responses, and that they do so in a carbon conditional manner that again reflects the carbon‐conditional sensitivities of *rtg3* and *sfp1* cells to these stresses (Figs. 8 and 9). Therefore, Rtg3 and Sfp1 appear to maintain key stress pathways and promote stress resistance under different growth conditions – Rtg3 during growth on lactate, and Sfp1 during growth on glucose (Fig. 10).

How does Rtg3 contribute to stress resistance during growth on lactate? In addition to promoting the induction of key stress regulators in lactate‐growing *C. albicans* cells (Figs. 8 and 9), Rtg3 appears to regulate the expression of a range of transporters, hydrolases and transcription factors, some of which may contribute to stress adaptation (Fig. 6). These include Hal21 (a phosphatase that is predicted to be involved in the hyperosmotic response) and Hac1 (a transcription factor that regulates the endoplasmic reticulum (ER) stress response in *C. albicans* (Wimalasena *et al*., 2008). They also include numerous mitochondrial functions (*COX3A, COX3B, COX4, TIM50*). In *S. cerevisiae*, Rtg3 is regulated by TOR signalling (Crespo *et al*., 2002) and contributes to mitochondrion‐to‐nucleus signalling via the retrograde response pathway (Rothermel *et al*., 1997; Jia *et al*., 1997; Jazwinski, 2014). While this type of intra‐organellar communication has not been studied extensively in *C. albicans*, mitochondrial functionality is known to influence stress resistance in this pathogenic yeast. For example, the inactivation of Goa1 (which is required for respiratory function and localizes to the mitochondrion under stress conditions) or Sam37 (a component of the mitochondrial outer membrane Sorting and Assembly Machinery complex) renders *C. albicans* cells sensitive to stresses, affects cell wall integrity and attenuates their virulence (*Jia et al*., 1997 *; Rothermel et al*., 1997; Bambach *et al*., 2009; Leach *et al*., 2012c; Qu *et al*., 2012; Yan *et al*., 2014). Also the attenuation of mitochondrial functionality by Rtg3 inactivation might reduce the ability of lactate‐growing cells to generate the metabolic energy required for stress adaptation.

How does Sfp1 enhance stress resistance during growth on glucose? In *S. cerevisiae*, Sfp1 is thought to be a downstream effector of the TORC kinase (Crespo *et al*., 2002; Jorgensen *et al*., 2004; Lempiäinen *et al*., 2009). Following activation via TORC signaling, Sfp1 activates a large number of *S. cerevisiae* genes (10%), mainly driving ribosomal protein synthesis and ribosome biogenesis under favorable nutrient conditions (Xu and Norris, 1998; Crespo *et al*., 2002; Jorgensen *et al*., 2004; Marion *et al*., 2004; Cipollina *et al*., 2008). Interestingly, in *C. albicans* Sfp1 appears to regulate a large number of ribosomal protein genes (*RPL5/8/20B/23A/28B/30/32/40B, RPS3/5/13/18/27*) (Fig. 6). In *S. cerevisiae*, the activity of the TORC kinase is low on lactate, compared to glucose. Also, TORC signaling has been shown to inhibit the retrograde response especially in the presence of glutamine (Dilova *et al*., 2002). Thus, the differential regulation of Sfp1 and Rtg3 by TORC signaling in response to carbon source might underlie the differential contributions of these transcription factors to stress resistance in *C. albicans*.

To summarize, this study has revealed that numerous regulators, in addition to the classical stress regulators, contribute to stress resistance of *C. albicans* cells under growth conditions that better reflect some of the key environmental inputs encountered in host niches. These genes were not previously identified using the standardized *in vitro* growth conditions that have generally been used in the past. Our analyses of two transcription factors, Rtg3 and Sfp1, have revealed mechanisms by which *C. albicans* retains stress resistance under different growth conditions and have highlighted the complexity of crosstalk between nutrient and stress signaling in this pathogen.

## Experimental procedures

### Strains and growth conditions

The mutant libraries used in the screens (Supporting Information Table S1), representing a total of 1158 strains, were generously provided by Suzanne Noble, Aaron Mitchell and Dominique Sanglard. *C. albicans rtg3 (ura3Δ/ura3Δ, his1Δ/his1Δ, arg4Δ/arg4Δ, rtg3::ARG4/rtg3*::*URA3)* and *sfp1* mutants *(ura3Δ/ura3Δ, his1Δ/his1Δ, arg4Δ/arg4Δ, orf19.5953::ARG4/orf19.5953::URA3)* (Vandeputte *et al*., 2011) were subjected to more detailed analysis. The genotypes of these strains were confirmed by diagnostic PCR using the primers described in Supporting Information Table S2. To confirm the phenotypes of the *rtg3* and *sfp1* mutants, the corresponding wild type gene was cloned into the vector CIp30 and integrated into the *RPS1* locus (Murad *et al*. 2000; Dennison *et al*., 2005). These reintegrant strains were compared with mutant strains transformed with the empty CIp30 vector to ensure that: (i) the histidine and arginine auxotrophies was repaired in both mutant and control and (ii) both mutant and control strains carried *URA3* at the *RPS1* locus to avoid *URA3‐*related position effects (Brand *et al*., 2004).

*C. albicans* were grown on YPD (2% glucose, 2% Mycopeptone, 1% yeast extract, 2% BactoAgar), GYNB (2% glucose, 0.67% yeast nitrogen base without amino acids, 2% BactoAgar), or LacYNB (2% sodium lactate, pH 7, 0.67% yeast nitrogen base without amino acids, 2% BactoAgar) containing uridine, histidine, arginine and leucine (400 μ/ml) at the specified temperatures (Sherman, 2002; Tillmann *et al*., 2011; You *et al*., 2012). To impose stress, 0.4 mM H_2_O_2_, 5 mM NaNO_2_ or 1 M NaCl were used *(*Chiranand *et al*., 2008 *;* Kaloriti *et al*., 2012). These concentrations were selected because in control experiments they were found to attenuate the growth of hallmark *cap1, cta4* and *hog1* strains, respectively, but not the wild type control strain.

To test cell viability, strains were grown on YPD at 30°C. Exponential phase cells were harvested, washed with YPD, GYNB or LacYNB, and then exposed to osmotic (1 M NaCl), oxidative (0.4 mM H_2_O_2_) or nitrosative stress (5 mM NaNO_2_ plus 25 mM succinic acid) or to no stress (control cells) for one hour. Cells were then washed using the same medium and left to recover for 120 min in the absence of stress. Percentage viability is presented, relative to the unstressed control. Mutants are compared with the corresponding re‐integrant strain using a 1‐way ANOVA ‐Dunnett's Multiple Comparison Test.

### Genetic screen

The 1158 *C. albicans* strains subjected to screening (Supporting Information Table S1) were organized into twelve 96‐well plates. Using a Singer RoToR robot (Singer Instruments, Watchet, UK), each strain was pinned (four spots per strain) onto plates prepared using RoToR Singer plates using the above media. Replicate plates were incubated for 24 h at 30°C, 37°C and 42°C and photographed using a GeneFlash (Syngene UK, Cambridge, UK) gel imager. Image analysis was conducted using Proteus pilot software, and growth assessed electronically via pixel quantification (0 = 0–20%; 1= 21–71%; 2 = 72–100% of normal growth under the same conditions except in the absence of stress). Each screen was performed in duplicate. A strain was defined as sensitive to a stress if it reproducibly displayed a > 80% decrease in growth with the stress, relative to no‐stress control plate with matching carbon source and temperature.

The screening output was validated by retesting the stress sensitivities of a selection of mutants using drop tests. To achieve this, strains were grown overnight in YPD at 30°C, subcultured into fresh YPD, and then grown at 30°C up to an OD_600_ of 1. Cells were then serially diluted, plated on to GYNB or LacYNB media containing the relevant stress, and then grown at the appropriate temperature.

### Data analysis and network visualization

The screening data generated by Proteus in Excel format were used to generate Venn Diagrams using Venny open source software (http://omictools.com/venny-s6319.html), and the output from these analyses was used to create filtered excel files that were subsequently analyzed through Cytoscape V3 (www.cytoscape.org/cy3.html) to construct biological networks. The analyses of GO terms was performed through the Term Finder tool of the *Candida* Genome Database (www.candidagenome.org). Significant differences of the input cluster in comparison to the background gene database were considered if the e‐value was < 0.001. Promoter analyses were performed via PatMatch in the *Candida* Genome Database. Positive hits contained at least one identical match to the consensus sequence of interest within the 5′‐region of annotated *C. albicans* genes. Graphs were constructed and statistical analyses performed using GraphPad Prism 6 software.

### Gene expression

Total RNA was isolated from exponentially growing *C. albicans* cells using the YeaStar^TM^ RNA Kit (Zymo Research, Irvine, U.S.A) according to the manufacturer's instructions. The RNA was treated with DNase I (Invitrogen, Paisley, UK) in the presence of RNase inhibitor (Rnase OUT: Invitrogen), and then the levels of specific transcripts subjected to qRT‐PCR using published procedures (Leach *et al*., 2012a). The primers are described in Supporting Information Table S2.

## Supporting information

Supporting Figure S1

Supporting Table S1

Supporting Table S2

Supporting Table S3
