# Supplementary material for: Sfp1 and Rtg3 reciprocally modulate carbon source‐conditional stress adaptation in the pathogenic yeast Candida albicans
Source: Mol Microbiol. 2017 Jun 19;105(4):620–36. doi: 10.1111/mmi.13722 (PMC5575477; doi:10.1111/mmi.13722)
Supplement: Supplementary file 1 — Supporting Figure S1 [file MMI-105-620-s001.pdf]

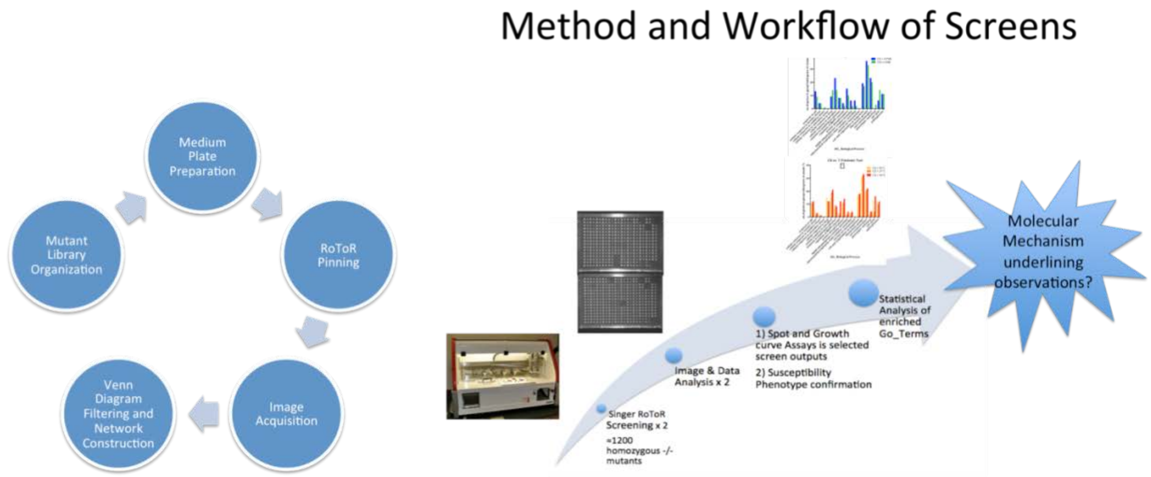

Figure S1. Flow chart of the screening strategy. Firstly, the five sets of mutants (Table S1) were combined into one library that incorporated the appropriate set of wild type parental strains (twelve 96-well plates in total). Secondly, using a Singer RoToR robot, strains were pinned (4 spots per strain) on to Singer plates containing the appropriate media and stressors (Materials and Methods). Each screen comprised 432 plates to allow parallel comparisons of the 12x96-well plate library on three carbon sources (YPD, glucose, lactate), at three different temperatures (30°C, 37°C, 42°C) with four stresses (control, cationic, oxidative, nitrosative). Thirdly, each screen was performed in duplicate to reduce false-positive discovery rate. Fourthly, each plate was imaged using a trans illuminator and the data was analyzed computationally using Proteus pilot software. The data were filtered to exclude mutants that failed to grow on the control plates. Fifthly, the data were then organized in excel files which were imported in Cytoscape software to generate networks.
