# Supplementary material for: Sfp1 and Rtg3 reciprocally modulate carbon source‐conditional stress adaptation in the pathogenic yeast Candida albicans
Source: Mol Microbiol. 2017 Jun 19;105(4):620–36. doi: 10.1111/mmi.13722 (PMC5575477; doi:10.1111/mmi.13722)
Supplement: Supplementary file 2 — Supporting Table S1 [file MMI-105-620-s002.pdf]

Table S1 Mutant libraries used in the genetic screens

Plate name	Provided by	No. of Mutants	Deletions (D) / Transposon Insertions (T)	Reference
CJN TFs	Mitchell, A.	83	(T)	(Nobile et al., 2009)
Kinases	Mitchell, A.	69	(D)	(Norice et al., 2007)
CWPs	Sanglard, D.	98	(D)	(Vandeputte et al., 2011)
TFs	Sanglard, D.	170	(T)	(Vandeputte et al., 2011)
MISC	Sanglard, D.	63	(T)	(Vandeputte et al., 2011)
NOBLE	Noble, S. M.	672	(D)	(Noble et al. 2010)
BRAND	Brand, A.	3	(D)	(Brand et al., 2008; Dunkel et al., 2008)
