# Supplementary material for: Sfp1 and Rtg3 reciprocally modulate carbon source‐conditional stress adaptation in the pathogenic yeast Candida albicans
Source: Mol Microbiol. 2017 Jun 19;105(4):620–36. doi: 10.1111/mmi.13722 (PMC5575477; doi:10.1111/mmi.13722)
Supplement: Supplementary file 3 — Supporting Table S2 [file MMI-105-620-s003.pdf]

Table S2 Diagnostic PCR primers for confirmation of *rtg3* and *sfp1* mutations

**PRIMER NAME**	**MUTANT**	**Sequence 5' to 3'**
F-RTG3	***rtg3Δ***	5' TTAAGGAAACTGAGGAGG
R-RTG3	***rtg3Δ***	5' CCACTGCCGGAGTAACCG
F-C3_04860W_A	***sfp1Δ***	5' GCAAAGAAAATAATTTGTTACG
R-C3_04860W_A	***sfp1Δ***	5' TACTTACTACTTGTTCAGGT
