# Supplementary material for: Sfp1 and Rtg3 reciprocally modulate carbon source‐conditional stress adaptation in the pathogenic yeast Candida albicans
Source: Mol Microbiol. 2017 Jun 19;105(4):620–36. doi: 10.1111/mmi.13722 (PMC5575477; doi:10.1111/mmi.13722)
Supplement: Supplementary file 4 — Supporting Table S3 [file MMI-105-620-s004.pdf]

Table S3 qRT-PCR Primers

**Primer Name**	**Transcript Examined**	**Sequence 5' to 3'**
L-NIK1	***NIK1***	5' GAGAAATTGCTGATGTCACACG
R-NIK1	***NIK1***	5' TGTACATTAATTTTACGTGACAAGTCC
L-YPD1	***YPD1***	5' AGAATTTGGAGAAATTGTCATCG
R-YPD1	***YPD1***	5' CGCATTGATTTGAAATTTTGG
L-SSK2	***SSK2***	5' TGGGAGCTAGATCAAGTGAGG
R-SSK2	***SSK2***	5' CTGGGAACTAGCAATATTATGACG
L-CTA4	***CTA4***	5' CTTATAGATCTCTTTATGGTCGAAAGG
R-CTA4	***CTA4***	5' GGATTTGTCGGATGTTGTAGG
L-CAT1	***CAT1***	5' CTACTTTGCTGAAGTTGAACAAGC
R-CAT1	***CAT1***	5' TGGATCAGCAGATGGTTCC
L-orf19.5953	***ORF19.5953***	5' CCAACAACAATCACCATCTAGC
R-orf19.5953	***ORF19.5953***	5' TCATCCTCGTCCGTTTGC
L-RTG3	***RTG3***	5' GCTGAGGATGACAAGAATAATTTAGC
R-RTG3	***RTG3***	5' AAGGAGGCACCACTAATGC
L-HOG1	***HOG1***	5' GATCACGTGCATCAATTCTCC
R-HOG1	***HOG1***	5' GTGTTTTCGGAACAAATAGTATC
